# A fine‐scale examination of parturition timing in temperate ungulates

**DOI:** 10.1002/ece3.11703

**Published:** 2024-07-03

**Authors:** Matthew T. Turnley, Tabitha A. Hughes, Randy T. Larsen, Kent R. Hersey, Matthew S. Broadway, M. Colter Chitwood, W. Sue Fairbanks, Robert C. Lonsinger, Brock R. McMillan

**Affiliations:** ^1^ Department of Natural Resource Ecology and Management Oklahoma State University Stillwater Oklahoma USA; ^2^ School of Natural Resources University of Nebraska‐Lincoln Lincoln Nebraska USA; ^3^ Department of Plant and Wildlife Sciences Brigham Young University Provo Utah USA; ^4^ Utah Division of Wildlife Resources Salt Lake City Utah USA; ^5^ U.S. Geological Survey, Oklahoma Cooperative Fish and Wildlife Research Unit Oklahoma State University Stillwater Oklahoma USA

**Keywords:** diel timing, elk, mule deer, parturition, phenology, survival

## Abstract

Parturition timing has long been a topic of interest in ungulate research. However, few studies have examined parturition timing at fine scale (e.g., <1 day). Predator activity and environmental conditions can vary considerably with diel timing, which may result in selective pressure for parturition to occur during diel times that maximize the likelihood of neonate survival. We monitored parturition events and early‐life survival of elk (*Cervus canadensis*) and mule deer (*Odocoileus hemionus*) in Utah, USA to better understand diel timing of parturition in temperate ungulates. Diel timing of parturition was moderately synchronous among conspecifics and influenced by environmental variables on the date of parturition. For elk, parturition events were most common during the morning crepuscular period and generally occurred later (i.e., closer to 12:00) when a relatively large proportion of the moon was illuminated. For mule deer, parturition events were most common during the diurnal period and generally occurred later (i.e., closer to 15:00) on cold, wet dates. Diel timing of parturition did not influence neonate survival, but larger datasets may be required to verify the apparent lack of influence. Although additional work could evaluate alternative variables that might affect parturition timing, our data provide an improved and finer scale understanding of reproductive ecology and phenology in ungulates.

## Graphical abstract

We monitored parturition events and early‐life survival of elk and mule deer in Utah, USA to better understand diel timing of parturition in temperate ungulates. Diel timing of parturition was moderately synchronous among conspecifics and influenced by environmental variables on the date of parturition.

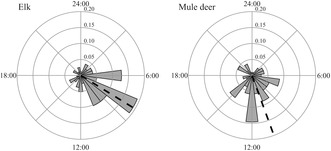

## 1 INTRODUCTION

Understanding parturition timing in ungulates requires a scale‐dependent perspective. On a broad scale (e.g., seasonally), parturition timing is primarily determined by vegetation quality. Parturition in temperate ungulates typically occurs in spring and early summer, coinciding with the nutrient‐rich period of new vegetation growth (English et al., 2012; Owen‐Smith & Ogutu, 2013; Stoner et al., 2016). Parturition in tropical ungulates tends to be most frequent during the wet season, when increased precipitation improves vegetation quality (Ogutu et al., 2013; Owen‐Smith & Ogutu, 2013; Sinclair et al., 2000). Late gestation and lactation are the most energetically demanding periods in a female ungulate's reproductive cycle, which likely drives the need for high‐quality nutrition (Gittleman & Thompson, 1988). On intermediate scales (e.g., daily or weekly within a season), parturition timing continues to be influenced by vegetation quality, but additional factors may also have an effect. For example, predation pressure on neonates can promote earlier and more synchronous parturition events (Patterson et al., 2016). Parturition synchrony with local conspecifics may lead to predator satiation while simultaneously being a response to peak vegetation quality (Michel et al., 2020; Rutberg, 1987). Maternal body condition further influences parturition timing. Females may fail to enter oestrus until sufficient body condition is achieved, resulting in delayed conception and parturition dates (Adams & Dale, 1998; Cook et al., 2004; Keech et al., 2000). Moreover, gestation may be lengthened for females in poor condition to allow for adequate foetal growth (Berger, 1992; Cameron et al., 1993). Consequently, correlates of female condition, including age, population density, precipitation, recent reproductive effort, and social rank have frequently been linked to parturition timing (Adams & Dale, 1998; Holand et al., 2004; Langvatn et al., 2004; Ogutu et al., 2015; Wolcott et al., 2015). A low male to female ratio or low mature male to female ratio may also result in delayed conception and parturition dates (Holand et al., 2003; Noyes et al., 1996; but see Freeman et al., 2014).

Despite the ecological significance of parturition timing at broad and intermediate scales, little is known about parturition timing in ungulates at fine scale (e.g., <1 day). It seems cogent that fine‐scale variations in parturition timing could be influenced by selective pressures and environmental conditions, especially considering such relationships are seen at broader scales. However, fine‐scale estimates of parturition timing in ungulates were limited until the advancement of vaginal implant transmitters (VITs), specialized devices expelled at parturition that can record the time of expulsion (Barbknecht et al., 2009; Johnstone‐Yellin et al., 2006). As a result, few studies have examined diel timing of parturition. Diel timing of parturition in temperate ungulates has been hypothesized to coincide with minimal predator activity, but this idea has not been thoroughly examined (Patterson et al., 2016). Predators of temperate ungulates tend to be crepuscular or nocturnal (Beier et al., 1995; Bridges et al., 2004; Thornton et al., 2004). Olfactory or visual stimuli associated with parturition may attract active predators, making neonates associated with crepuscular or nocturnal parturition events more susceptible to predation. Consequently, factors that influence diel activity patterns of predators may indirectly influence diel timing of parturition. For example, visually oriented predators often increase crepuscular and nocturnal activity during lunar phases with more illumination, potentially linking moon illumination and diel timing of parturition (Botts et al., 2020; Melville et al., 2020; Prugh & Golden, 2014). Diel timing of parturition in temperate ungulates has also been hypothesized to coincide with relatively warm temperatures (Patterson et al., 2016). Severe weather can cause considerable reductions in neonate survival, especially cold and wet weather (Gilbert & Raedeke, 2004; Grovenburg et al., 2012). Nevertheless, a thorough investigation of the relationship between temperature and diel timing of parturition has not been performed.

Variations in parturition timing at broad and intermediate scales can have major fitness consequences for ungulates. In species that reproduce year‐round, season of parturition can influence neonate survival (Chinn et al., 2021; Lee et al., 2017). Unsurprisingly, neonate survival is typically higher in seasons where selected forage peaks in quality (Lee et al., 2017; Sinclair et al., 2000). Parturition timing can also influence neonate survival within a season. In some systems, neonate survival is highest during the peak parturition period, supporting the predator satiation hypothesis (Adams et al., 1995; Gregg et al., 2001; Michel et al., 2020). In other systems, neonate survival is inversely related to parturition date (Festa‐Bianchet, 1988; Lamb et al., 2023; Smith & Anderson, 1998; Testa et al., 2000). It is unclear why different patterns occur, but relatively late parturition events are almost always associated with reduced neonate survival. Neonates born relatively late in a season may be exposed to more efficient predators and have less time to gain mass before vegetation quality declines (Testa, 2002). Body mass at the onset of winter is often the best predictor of over‐winter survival for neonates in temperate systems, with relatively heavy individuals having a higher likelihood of survival than lighter individuals (Cook et al., 2004; Lamb et al., 2023; Taillon et al., 2006). Parturition timing can also have consequences for future reproduction; females with relatively late parturition events may be less likely to become pregnant the following year (Clutton‐Brock et al., 1983; Green & Rothstein, 1993). No previous study has examined the influence of diel timing of parturition on survival or future reproduction. Thus, the causes and consequences of diel timing of parturition remain largely unknown.

Our objectives were to better understand which factors (if any) influenced diel timing of parturition and if diel timing of parturition influenced neonate survival in temperate ungulates. We used elk (*Cervus canadensis*) and mule deer (*Odocoileus hemionus*) as model organisms. For both species, we hypothesized diel timing of parturition would not be uniformly distributed. We predicted parturition would be most common during the diurnal period when many temperate predators are less active (Patterson et al., 2016). We also hypothesized diel timing of parturition would be influenced by environmental variables on the date of parturition. We predicted there would be a positive relationship between moon illumination and the likelihood of parturition occurring during the diurnal period, particularly on nights with relatively little cloud cover. We predicted there would be a negative relationship between mean temperature on the date of parturition and the likelihood of parturition occurring during the diurnal period. In addition, we predicted there would be a positive relationship between total precipitation on the date of parturition and the likelihood of parturition occurring during the diurnal period. Finally, we hypothesized diel timing of parturition would influence neonate survival. We predicted survival would be higher for neonates associated with diurnal parturition events than for neonates associated with crepuscular or nocturnal parturition events.

## 2 MATERIALS AND METHODS

### 2.1 Study area

**FIGURE 1:**
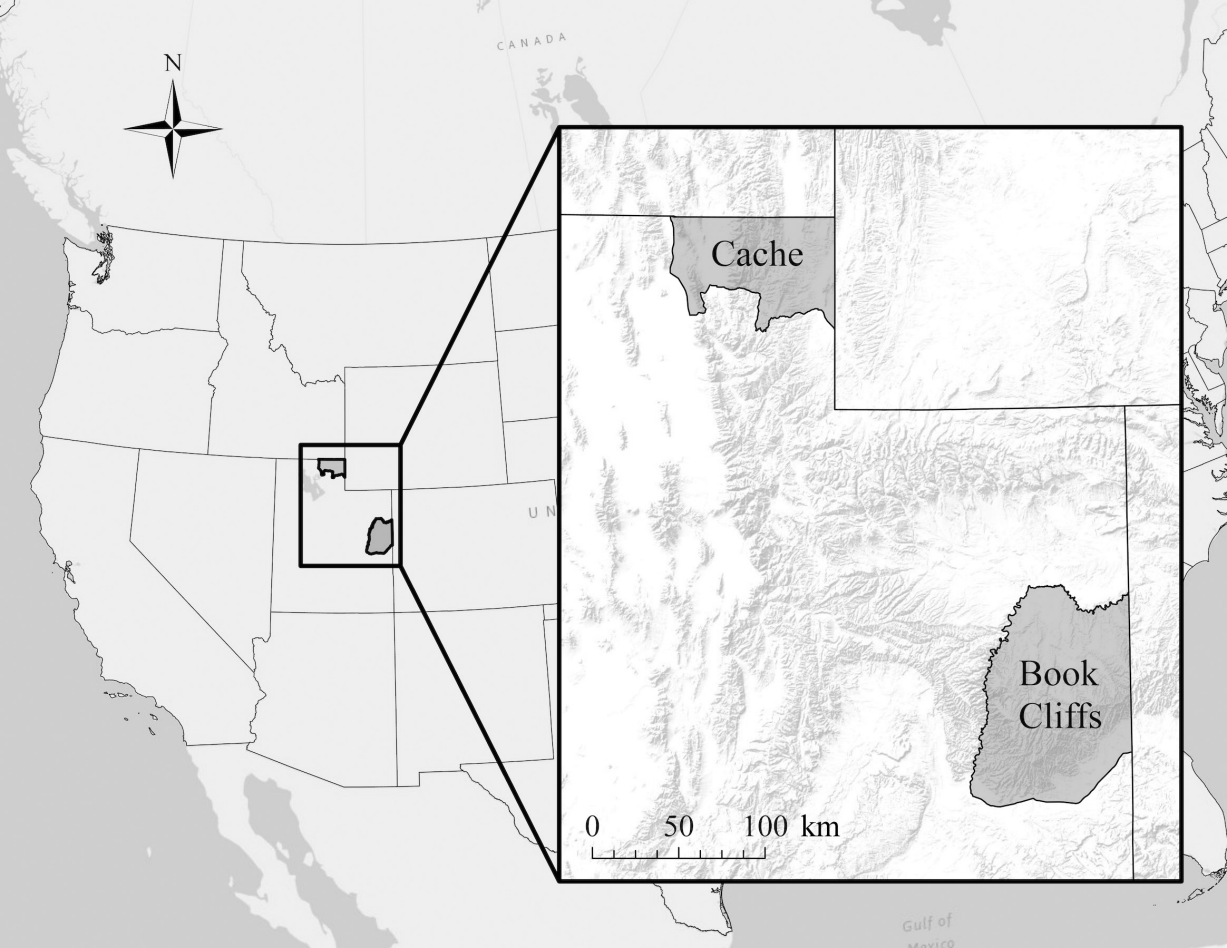
Study areas associated with parturition events for elk and mule deer during 2018–2021 in Utah, USA. All parturition events for elk occurred in the Book Cliffs management unit (*n* = 78), whereas parturition events for mule deer occurred in the Book Cliffs (*n* = 44) and Cache management units (*n* = 59).

We conducted this study in the Book Cliffs (39.5°, −109.3°) and Cache (41.7°, −111.5°) management units of Utah, USA (Figure 1). In the Book Cliffs, elevations ranged from 1675 to 2590 m over an area of ~9300 km^2^. Terrain included cliff faces, ridges, valleys, and flatlands. Mean annual temperature was 8.4°C and mean annual precipitation was 21.8 cm (30‐year means, 1991–2020; PRISM Climate Group, 2022). Common vegetation communities were sagebrush steppes (*Artemisia* spp.) and pine‐juniper woodlands (*Pinus monophyla*–*Juniperus oteosperma*). In the Cache, elevations ranged from 1300 to 3040 m over an area of ~4000 km^2^. Terrain included ridges and valleys. Mean annual temperature was 8.5°C and mean annual precipitation was 43.7 cm (30‐year means, 1991–2020; PRISM Climate Group, 2022). Common vegetation communities were sagebrush steppes and pine‐aspen woodlands (*Pseudotsuga menziesii*–*Populus tremuloides*). In the Book Cliffs and the Cache, potential predators of ungulates included black bears (*Ursus americanus*), bobcats (*Lynx rufus*), coyotes (*Canis latrans*), and mountain lions (*Puma concolor*).

### 2.2 Adult capture

We captured adult female elk and mule deer 3–5 months prior to the predicted parturition period to allow for efficient monitoring of parturition events. We captured elk in the Book Cliffs during February of 2019–2021 using helicopter net‐gunning or darting (Barrett et al., 1982; Krausman et al., 1985; McCorquodale et al., 1988). For dart captures, we used barbed darts (type U, 1.5 mL; Pneudart, Williamsport, PA, USA) equipped with thiafentanil (10 mg; Wildlife Pharmaceuticals, Laramie, WY, USA) and xylazine (30 mg; Wildlife Pharmaceuticals) to induce immobilization. We administered naltrexone (200 mg; Wildlife Pharmaceuticals) and atipamezole (10 mg; Wildlife Pharmaceuticals) to reverse immobilization. We captured mule deer in the Book Cliffs and the Cache during February–March of 2018–2021 using helicopter net‐gunning (Barrett et al., 1982; Krausman et al., 1985). We performed all captures with assistance from an independent capture company (Helicopter Wildlife Services, Austin, TX, USA or Quicksilver Air Inc., Peyton, CO, USA) and the Utah Division of Wildlife Resources (Salt Lake City, UT, USA).

Upon capture, handling protocols were similar for both species. We restrained and blindfolded non‐immobilized individuals (i.e., captured via net‐gunning) and fitted each with a radio collar (G5‐2DH, 657 or 595 g; Advanced Telemetry Systems, Isanti, MN, USA). Radio collars were equipped with global positioning system (GPS) technology and emitted very high frequency (VHF) transmissions. We determined the pregnancy status of each individual using transabdominal ultrasonography (Bishop et al., 2007; Stephenson et al., 1995). We inserted a VIT (M3960U: 41 g or M3930U: 23 g; Advanced Telemetry Systems) into pregnant individuals using a vaginoscope (Bishop et al., 2007, 2011).

### 2.3 Parturition time and environmental conditions

The VITs we deployed included light and temperature sensors to detect expulsion (i.e., parturition), which was indicated by an increase in light exposure or a temperature <32°C. An ultra high frequency (UHF) link between each female's collar and VIT allowed collars to record the time of parturition, the location of parturition, and to send notification of parturition via email. We used the temporal and spatial data associated with each parturition event when obtaining environmental data related to each parturition date. Environmental variables included proportion of the moon illuminated (a quantitative description of moon phase), mean cloud cover during the nocturnal period (22:00–3:59), mean temperature (2 m above ground), and total precipitation on the date of parturition. We obtained all environmental data from independent databases (Astronomical Applications Department of the U.S. Naval Observatory, 2023; Zippenfenig, 2023).

### 2.4 Neonate capture and survival

We captured neonatal elk and mule deer during May–July of 2019–2021 and May–June of 2018–2021, respectively. We waited >3 h following parturition before locating neonates, allowing time for mother–neonate bonding, cleaning of the neonate, and other post‐parturition events to occur (Haskell et al., 2007; Turnley et al., 2022). We located neonates by systematically searching the area surrounding an expelled VIT and (if needed) the female's recent coordinates. Upon capture, we blindfolded each neonate and wore nitrile gloves while processing. We fitted each neonate with an expandable VHF radio collar (M4230BU, 125 g; Advanced Telemetry Systems).

We monitored the survival of each neonate for 1 week following parturition—we did not expect diel timing of parturition to influence survival over a longer period. A UHF link between radio collars on each neonate and mother allowed for notification of mortality if a neonate's collar did not move for ≥4 h. Following a mortality notification, we located the collar and classified a neonate as deceased only if a carcass (or part of a carcass) was present. We generally located neonates within 24 h of receiving the mortality notification and located all neonates within 48 h of receiving the mortality notification. When possible, we also determined the cause of mortality based on characteristic patterns associated with various forms of mortality (Stonehouse et al., 2016). For example, hemorrhaging near tooth punctures and signs of a struggle indicated predation, while an empty stomach and lack of mortal injuries indicated maternal abandonment, particularly if the mother's recent GPS coordinates were not near the neonate (Livezey, 1990; Stonehouse et al., 2016). Animal handling and monitoring procedures were approved by the Institutional Animal Care and Use Committee at Brigham Young University (protocol 19‐0202) and in compliance with guidelines from the American Society of Mammalogists (Sikes & Animal Care and Use Committee of the American Society of Mammalogists, 2016).

### 2.5 Data analysis

We analyzed data using R version 4.3.1 (R Core Team, 2023). Considering the circular nature of diel timing (i.e., start and end points are synonymous), we performed all analyses using statistical methods appropriate for circular data and converted parturition time to radians prior to analysis (Landler et al., 2018; Lee, 2010). Notably, we analyzed diel timing of parturition as a continuous variable, but used four diel periods when categorical descriptors improved the clarity of our interpretations: morning crepuscular (4:00–9:59), diurnal (10:00–15:59), evening crepuscular (16:00–21:59), and nocturnal (22:00–3:59; Cochrane et al., 2019; Gese et al., 2016; Lewis & Rachlow, 2011). We used Rayleigh's test of uniformity to determine if diel timing of parturition was uniformly distributed for elk or mule deer (CircStats package; Lund & Agostinelli, 2018). We used Watson's test of homogeneity to determine if diel timing of parturition differed between elk and mule deer. Similarly, we used Watson's test of homogeneity to determine if diel timing of parturition for mule deer differed between the Book Cliffs and the Cache (circular package; Agostinelli & Lund, 2023). We used generalized additive mixed models (GAMMs) in a model selection framework to examine the relationship between diel timing of parturition and environmental conditions (mgcv package; Wood, 2004). Environmental variables included proportion of the moon illuminated, mean cloud cover during the nocturnal period, mean temperature, and total precipitation on the date of parturition. We constructed 11 a priori GAMMs for each species and modeled all predictor variables with cyclic cubic splines to allow for potential nonlinear effects. In addition, we included individual, year, and management unit (mule deer only) as random effects. We constructed models of elk parturition with a gamma error distribution and models of mule deer parturition with a Gaussian error distribution, as dictated by goodness‐of‐fit estimation. To select the most‐supported model, we used Akaike's Information Criterion adjusted for small sample sizes (AICc; Burnham & Anderson, 2002). We considered models with a ΔAICc <2.0 and without uninformative parameters to be competitive (Arnold, 2010; Leroux, 2019).

We used a GAMM constructed with a binomial error distribution to examine the relationship between diel timing of parturition and neonate survival to 1 week (coded as survived = 0, did not survive = 1) for mule deer; we did not have a sufficient sample size of mortalities to analyze survival of elk (mgcv package; Wood, 2004). We modeled parturition time with a cyclic cubic spline to allow for potential nonlinear effects. We included maternal identity, year, and management unit as random effects. We did not include additional fixed or random effects due to the limited sample size of neonate mortalities (*n* = 30). Moreover, we censored stillbirths (*n* = 10) and capture‐related mortalities (*n* = 2) from our survival analysis to limit confounding influences on neonate survival. For all analyses, we confirmed that relevant model assumptions were met and based interpretations on an *α*‐value of .05 (Guisan et al., 2002).

## 3 RESULTS

### 3.1 Influences on diel timing of parturition

**FIGURE 2:**
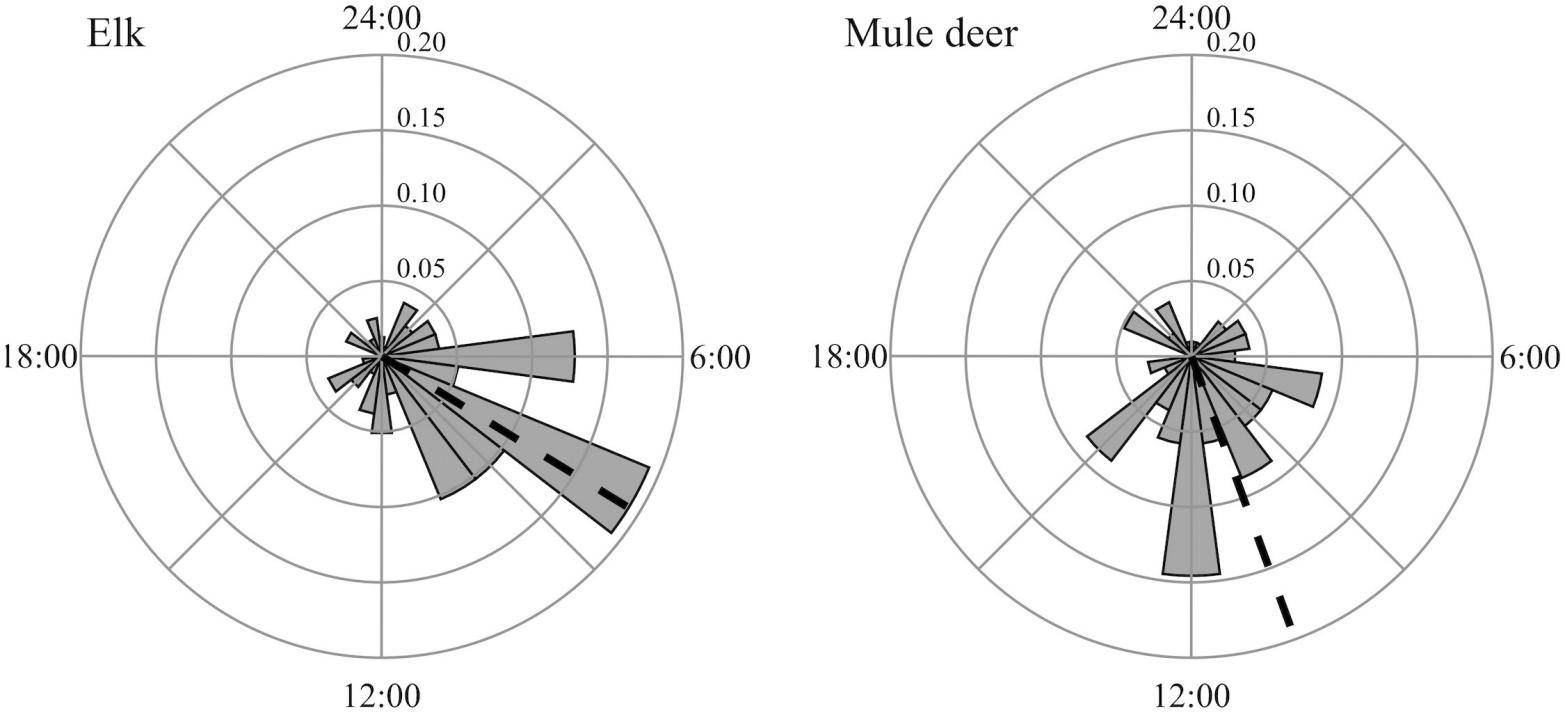
Diel timing of parturition for elk and mule deer during 2018–2021 in Utah, USA. Concentric circles indicate proportion of parturition events (i.e., relative frequency); proportion increases as circle size increases. Each gray bin represents a 1‐h period. Dashed lines represent circular means.

We analyzed data from 181 parturition events (78 elk, 103 mule deer) over a period of 4 years. Diel timing of parturition was not uniformly distributed for elk (*z* = 23.96, *p* ≤ .001, *r* = .55) or mule deer (*z* = 17.12, *p* ≤ .001, *r* = .41; Figure 2). Mean time of parturition for elk was 08:06 (SD = 4:09), with 45 (57.7%) parturition events occurring during the morning crepuscular period, 19 (24.4%) during the diurnal period, 4 (5.1%) during the evening crepuscular period, and 10 (12.8%) during the nocturnal period. Elk had earlier parturition events than mule deer (*w* = 0.40, *p* < .001). Mean time of parturition for mule deer was 10:39 (SD = 5:07), with 34 (33.0%) parturition events occurring during the morning crepuscular period, 46 (44.7%) during the diurnal period, 14 (13.6%) during the evening crepuscular period, and 9 (8.7%) during the nocturnal period. Diel timing of parturition for mule deer in the Book Cliffs (*n* = 44 parturition events) and the Cache (*n* = 59 parturition events) were similar (*w* = 0.07, *p* > .10).

**FIGURE 3:**
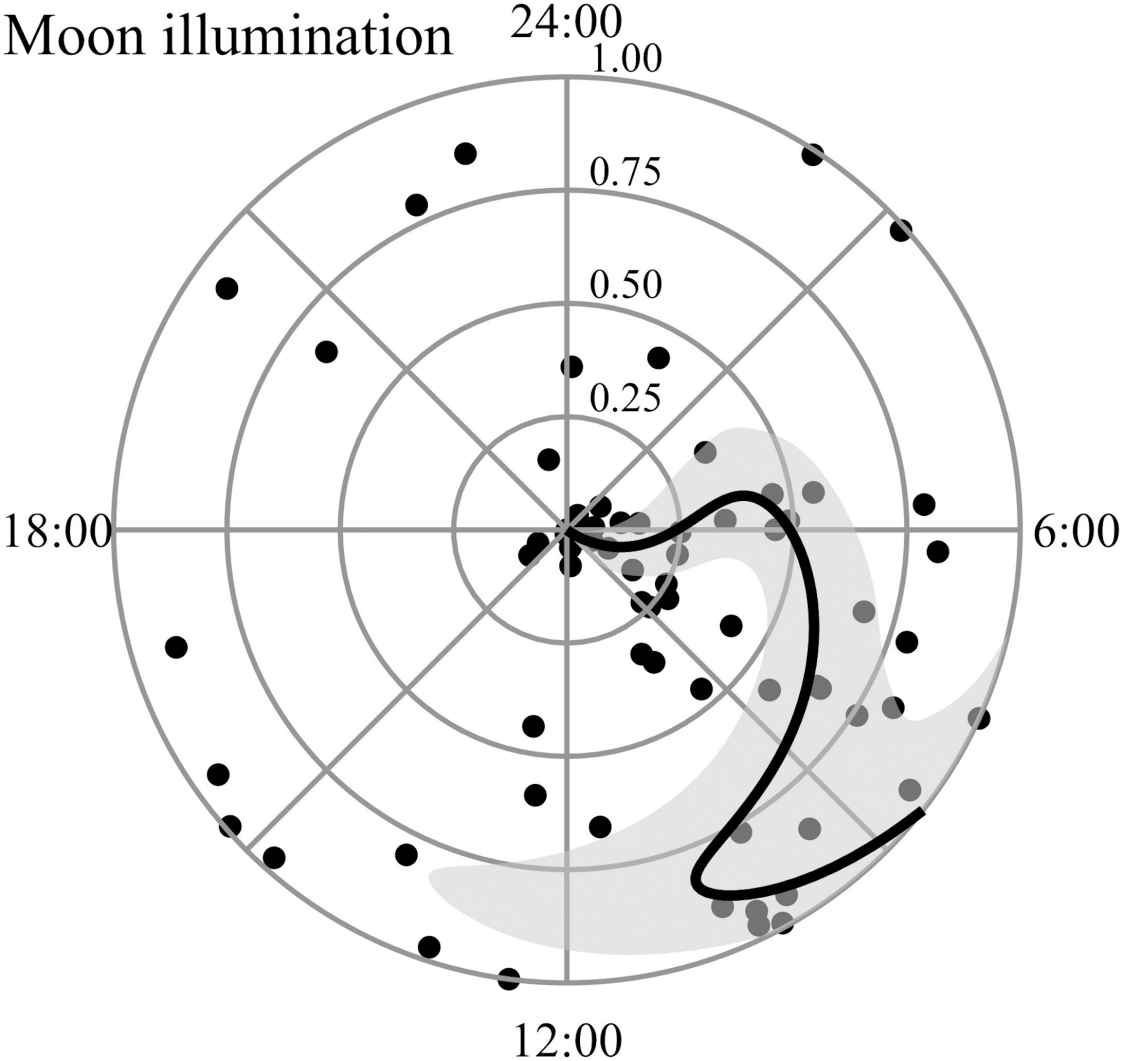
Predicted parturition times based on the environmental variable that influenced diel timing of parturition for elk: Moon illumination on the date of parturition. Concentric circles indicate proportion of the moon illuminated; illumination increases as circle size increases. Dots indicate recorded parturition times. Shaded area represents a 95% confidence interval for the predictive line. All parturition events occurred during 2019–2021 in Utah, USA.

The most‐supported model of diel timing of parturition for elk included proportion of the moon illuminated as its only parameter (*F* = 2.27, *p* ≤ .001, edf = 2.72, *R* ^2^ = .08; Table 1). All models that included moon illumination outperformed models without moon illumination. Although the relationship between moon illumination and diel timing of parturition for elk was nonlinear, predicted parturition times when >60% of the moon was illuminated were approximately equal to or later than predicted parturition times when <60% of the moon was illuminated. Predicted parturition times based on moon illumination ranged from 5:15 to 10:40 (Figure 3). The only other models with a ΔAICc <2.0 included mean temperature and nocturnal cloud cover on the date of parturition as additive parameters to moon illumination, respectively. However, there was little improvement in log likelihood or deviance from the most‐supported model, indicating temperature and cloud cover were uninformative (Arnold, 2010; Leroux, 2019).

**TABLE 1:** Model selection results for models of elk (*Cervus canadensis*) diel timing of parturition as a function of environmental conditions on the date of parturition.

Model	*K*	ΔAICc	*w*_*i*_	LogL	Dev
s(Moon)	4	0.00	0.32	−76.92	153.83
s(Moon) + s(Temp)	5	0.58	0.24	−76.21	152.41
s(Moon) + s(Cloud)	5	1.36	0.16	−76.60	153.19
s(Moon) + s(Temp) + s(Precip)	6	2.71	0.08	−76.27	152.54
s(Moon) + s(Precip)	5	2.79	0.08	−77.31	154.63
s(Moon, Cloud) + s(Moon) + s(Cloud)	7	3.63	0.05	−75.73	151.46
s(Temp)	4	5.15	0.02	−79.49	158.99
Null	3	5.99	0.02	−80.91	161.82
s(Temp) + s(Precip)	5	7.62	0.01	−79.73	159.45
s(Precip)	4	8.69	0.00	−81.26	162.52
s(Temp, Precip) + s(Temp) + s(Precip)	7	11.62	0.00	−79.73	159.45

*Note*: Environmental variables included proportion of the moon illuminated (Moon), mean temperature (Temp), mean cloud cover during the nocturnal period (Cloud), and total precipitation (Precip). All predictor variables were modeled with cyclic cubic splines (s). The null model included the intercept and random effects for individual and year, but did not include environmental variables. Ranking order determined by Akaike's Information Criterion adjusted for small sample sizes (AICc). Each model includes the number of estimated parameters (*K*), difference in AICc from the top model (ΔAICc), model weight (*w*_*i*_), log likelihood (LogL), and deviance (Dev). All parturition events occurred during 2019–2021 in Utah, USA.

**FIGURE 4:**
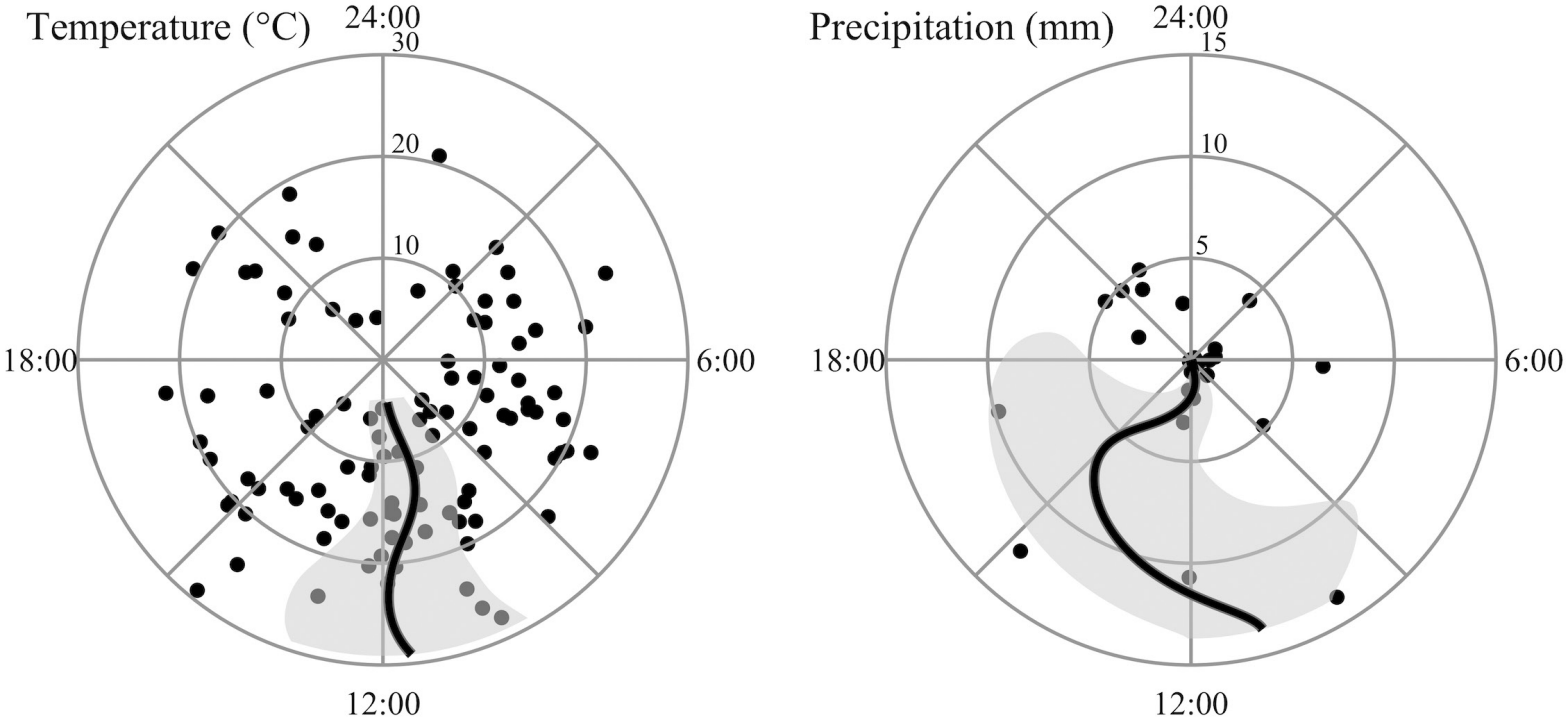
Predicted parturition times based on the environmental variables that influenced diel timing of parturition for mule deer: Temperature and precipitation. Concentric circles indicate mean temperature and total precipitation on the date of parturition, respectively; temperature and precipitation increase as circle size increases. Dots indicate recorded parturition times. Shaded areas represent 95% confidence intervals for the predictive lines. All parturition events occurred during 2018–2021 in Utah, USA.

The most‐supported model to explain diel timing of parturition for mule deer included temperature (*F* = 0.75, *p* = .03, edf = 1.89) and precipitation (*F* = 1.25, *p* = .003, edf = 1.87) as additive parameters (*R* ^2^ = .11; Table 2). The model that included only precipitation (*F* = 0.74, *p* = .02, edf = 1.51, *R* ^2^ = .06) and the null model (i.e., intercept and random effects only) were also competitive. Precipitation was included in four of the five highest ranking models. The model that included moon illumination as an additive parameter to precipitation had a ΔAICc <2.0, but there was little improvement in log likelihood or deviance from the precipitation‐only model, indicating moon illumination was uninformative (Arnold, 2010; Leroux, 2019). The relationship between temperature and diel timing of parturition for mule deer was predominantly negative when mean temperature was <15°C and predominantly positive when mean temperature was >15°C. Predicted parturition times based on mean temperature ranged from 11:00 to 11:55 (Figure 4). The relationship between precipitation and diel timing of parturition for mule deer was nonlinear, but predicted parturition times were generally later on dates with precipitation than on dates without precipitation. Predicted parturition times based on total precipitation ranged from 11:00 to 14:58 (Figure 4).

**TABLE 2:** Model selection results for models of mule deer (*Odocoileus hemionus*) diel timing of parturition as a function of environmental conditions on the date of parturition.

Model	*K*	ΔAICc	*w*_*i*_	LogL	Dev
s(Temp) + s(Precip)	6	0.00	0.26	−172.29	344.58
s(Precip)	5	0.36	0.22	−173.47	346.93
Null	4	1.61	0.12	−175.09	350.18
s(Moon) + s(Precip)	6	1.88	0.10	−173.23	346.46
s(Moon) + s(Temp) + s(Precip)	7	2.00	0.10	−172.29	344.58
s(Moon)	5	3.01	0.06	−174.79	349.59
s(Temp, Precip) + s(Temp) + s(Precip)	8	3.06	0.06	−171.82	343.63
s(Temp)	5	3.42	0.05	−175.00	350.00
s(Moon) + s(Temp)	6	5.01	0.02	−174.79	349.59
s(Moon) + s(Cloud)	6	5.01	0.02	−174.79	349.59
s(Moon, Cloud) + s(Moon) + s(Cloud)	8	8.97	0.00	−174.77	349.55

*Note*: Environmental variables included proportion of the moon illuminated (Moon), mean temperature (Temp), mean cloud cover during the nocturnal period (Cloud), and total precipitation (Precip). All predictor variables were modeled with cyclic cubic splines (s). The null model included the intercept and random effects for individual, year, and management unit, but did not include environmental variables. Ranking order determined by Akaike's information criterion adjusted for small sample sizes (AICc). Each model includes the number of estimated parameters (*K*), difference in AICc from the top model (ΔAICc), model weight (*w*_*i*_), log likelihood (LogL), and deviance (Dev). All parturition events occurred during 2018–2021 in Utah, USA.

### 3.2 Consequences of diel timing of parturition

We captured 231 neonates (77 elk, 154 mule deer) and monitored their survival for 1 week post‐parturition. There were nine (11.7%) elk that died within a week of parturition, with mortalities attributed to predation (7; 77.8%), accidental injury (1; 11.1%), or unknown causes (1; 11.1%). There were 30 (19.5%) mule deer that died within a week of parturition, with mortalities attributed to unknown causes (18; 60.0%), predation (10; 33.3%), accidental injury (1; 3.3%), or maternal abandonment (1; 3.3%). The limited number of elk mortalities within a week of parturition (*n* = 9) precluded our analysis of the relationship between diel timing of parturition and survival for elk. There was no relationship between diel timing of parturition and survival for mule deer (*χ* ^2^ < 0.001, *p* = .58).

## 4 DISCUSSION

Our results indicated diel timing of parturition was moderately synchronous among conspecifics. Parturition events for elk were most common during the morning crepuscular period, while parturition events for mule deer were most common during the diurnal period. The two previous examinations of diel timing of parturition in ungulates detected analogous patterns. Parturition events for wildebeest (*Connochaetes taurinus*) were most common during the morning crepuscular period, while parturition events for moose (*Alces alces*) were most common during the diurnal period (Estes & Estes, 1979; Patterson et al., 2016). We did not attempt to elucidate why parturition timing differed between elk and mule deer, but our results provide an improved understanding of variables that influence diel timing of parturition.

Parturition events for elk generally occurred later (i.e., closer to 12:00) when a relatively large proportion (i.e., >60%) of the moon was illuminated than when a relatively small proportion of the moon was illuminated. It seems plausible this response was a predator‐avoidance strategy, which partially supported the idea that parturition may coincide with reduced predator activity (Patterson et al., 2016). Coyotes, mountain lions, and other visually oriented predators tend to increase crepuscular and nocturnal activity during bright lunar periods because increased moonlight facilitates prey detection (Botts et al., 2020; Melville et al., 2020; Prugh & Golden, 2014). Although our study system included predators with a variety of sensory systems and hunting modes, delayed parturition events during bright lunar periods may have limited the temporal overlap between parturition and active predators. Nevertheless, it remains unclear why parturition events for elk were not more common during the diurnal period regardless of moon illumination. The relative frequency of parturition events during the morning crepuscular period provided little support for the idea that parturition coincides with reduced predator activity. In addition, it remains unclear why the relationship between parturition timing and moon illumination was not influenced by nocturnal cloud cover. Cloud cover often mediates the influence of moon illumination on other aspects of ungulate behavior, including antipredator behavior (Cerri et al., 2023; Ladine & Settles, 2020). Parturition events for mule deer were more diurnal than crepuscular, which may explain why we did not observe relationships between moon illumination, nocturnal cloud cover, and parturition timing for mule deer.

Thermal characteristics associated with the date of parturition influenced diel timing of parturition for mule deer. Parturition timing shifted (albeit only slightly) to limit the temporal overlap between parturition and relatively cold temperatures but did not limit the temporal overlap between parturition and relatively warm temperatures. Animals with a larger surface area to volume ratio typically have greater heat dissipation than animals with a smaller surface area to volume ratio, which may explain the evidence for mule deer being more cold‐sensitive than elk (Porter & Kearney, 2009). Parturition events for mule deer also tended to occur later (i.e., closer to 15:00) on dates with precipitation than on dates without precipitation. Our precipitation‐related results should be interpreted with caution considering the small sample of parturition dates with substantial precipitation (*n* = 12 dates with >3.0 mm), but the observed relationship further suggested parturition timing of mule deer shifted to limit cold stress. Exposure to precipitation increases heat dissipation in ungulates, especially in neonates and adults with relatively thin summer pelage (Parker, 1998). Thermal stress associated with cold temperatures can result in fitness consequences for ungulates, increasing energetic requirements and potentially decreasing neonate survival under extreme circumstances (Gilbert & Raedeke, 2004; Grovenburg et al., 2012; Linnell et al., 1995; Parker, 1998). To reduce thermal stress at parturition, females often select parturition sites with high thermal cover (Barbknecht et al., 2011; Bowyer et al., 1999; Etchberger & Krausman, 1999). Moreover, neonates select bed sites with high thermal cover and may adjust bed site selection based on ambient temperature (Bowyer et al., 1998; Grovenburg et al., 2012; Huegel et al., 1986; Kjellander et al., 2012). Our results provide evidence of a temporal response within the diel period reducing thermal stress at parturition in addition to the better‐known spatial responses. We emphasize our study was not designed to determine if shifts in parturition timing were the result of female choice or involuntary physiological mechanisms. Multiple hormonal changes are associated with parturition in mammals, but the proximate triggers of these hormonal changes are not fully understood (Honnebier & Nathanielsz, 1994; Nathanielsz, 1998).

Diel timing of parturition did not influence survival for neonatal mule deer. The lack of a temporal influence on survival was surprising considering diel timing of parturition was moderately synchronous. When parturition synchrony is displayed at broader scales, neonates born during the peak parturition period often have higher survival than neonates born outside the peak parturition period (Adams et al., 1995; Gregg et al., 2001; Michel et al., 2020). If survival of neonatal mule deer was really independent of diel timing of parturition, then parturition may not have attracted predators (which were generally crepuscular/nocturnal) to the extent we originally hypothesized. However, we caution that our survival analysis was limited. Only 23 (22.3%) parturition events for mule deer occurred during the evening crepuscular period or the nocturnal period, limiting the temporal variation in our analysis. Moreover, only 30 (19.5%) neonatal mule deer died within our 1‐week monitoring period, restricting the number of confounding variables we were able to include as random effects. For example, we could not account for birth weight, litter size, or parturition date in our survival analysis, all of which can influence survival of neonatal ungulates (Chitwood et al., 2015; Johnstone‐Yellin et al., 2009; Kilgo et al., 2012; Linnell et al., 1995; Lomas & Bender, 2007). We present our survival‐related result not as a concrete conclusion but as a foundation for further research (Bissonette, 1999). Although more investigation is needed, we offer a rare, fine‐scale examination of parturition timing, and an improved understanding of reproductive ecology in ungulates.

## AUTHOR CONTRIBUTIONS

**Matthew T. Turnley:** Formal analysis (equal); investigation (equal); writing – original draft (lead); writing – review and editing (equal). **Tabitha A. Hughes:** Formal analysis (equal); investigation (equal); writing – review and editing (equal). **Randy T. Larsen:** Conceptualization (equal); investigation (equal); writing – review and editing (equal). **Kent R. Hersey:** Conceptualization (equal); investigation (equal); writing – review and editing (equal). **Matthew S. Broadway:** Formal analysis (equal); writing – review and editing (equal). **M. Colter Chitwood:** Formal analysis (equal); writing – review and editing (equal). **W. Sue Fairbanks:** Formal analysis (equal); writing – review and editing (equal). **Robert C. Lonsinger:** Formal analysis (equal); writing – review and editing (equal). **Brock R. McMillan:** Conceptualization (equal); investigation (equal); writing – review and editing (equal).

## CONFLICT OF INTEREST STATEMENT

We have no conflicts of interest to report.

## Data Availability

Data, metadata, and code are available in the Figshare digital repository: https://doi.org/10.6084/m9.figshare.c.7081711 (Turnley et al., 2024).
